# Multiscale detection of power quality disturbances and cyber intrusions in smart grids using NSCT and frequency band scalograms

**DOI:** 10.1038/s41598-025-18127-2

**Published:** 2025-09-05

**Authors:** Pampa Sinha, Kaushik Paul, Snehalika Snehalika, Idamkati Kasireddy, Ardhala Bala Krishna, D. S. Naga Malleswara Rao, Hassan Abdurrahman Shuaibu, Taha Selim Ustun

**Affiliations:** 1 KIIT University, Bhubaneswar, India; 2 BIT Sindri, Dhanbad, India; 3 Vishnu Institute of Technology, Bhimavaram, 534202 India; 4 Potti Sriramulu Chalavadi Mallikarjuna Rao College of Engineering and Technology, Vijayawada, India; 5 Gokaraju Rangaraju Institute of Engineering & Technology Hyderabad, Telangana, 500090 India; 6 Department of Electrical, Telecommunications and Computer Engineering, Kampala International University, Kampala, Uganda; 7 Fukushima Renewable Energy Institute, Koriyama, Japan

**Keywords:** Power quality (PQ), Nonsubsampled contourlet transform (NSCT), Morphological component analysis (MCA), Split augmented lagrangian shrinkage approach (SALSA), Frequency-Adaptive detection index (FADI), Cyber-physical security, False data injection (FDI), Denial of service (DoS), Multi-Class support vector machine (SVM), Smart grids, Energy infrastructure, Electrical and electronic engineering

## Abstract

This paper presents a novel multiscale signal processing framework for power quality disturbance (PQD) and cyber intrusion detection in smart grids, combining Non-Subsampled Contourlet Transform (NSCT), Split Augmented Lagrangian Shrinkage Algorithm (SALSA), and Morphological Component Analysis (MCA). A key innovation lies in an adaptive weighting mechanism within NSCT’s directional sub bands, enabling dynamic energy redistribution and enhanced representation of both low-frequency anomalies (e.g., voltage sags/swells) and high-frequency distortions (e.g., harmonics, transients). SALSA-based sparse optimization achieves an average signal-to-noise ratio (SNR) improvement of 12.8 dB, preserving essential transient structures, while MCA isolates fault-relevant morphological components for better interpretability. Extensive simulations on both synthetic signals and the IEEE 14-bus test system demonstrate detection accuracies of 98.6% for PQDs and 97.2% for cyber intrusions, including False Data Injection (FDI), Denial of Service (DoS), and Command Injection attacks. Each intrusion exhibits unique time-frequency scalogram signatures, which are effectively visualized using high-resolution, denoised 2D/3D spectrograms generated via adaptive Q-Factor Wavelet Transform (AQWT) and Short-Time Fourier Transform (STFT). Compared to baseline methods like STFT-only and DWT-SVM pipelines, the proposed NSCT-SALSA-MCA framework improves detection precision by 14–18%, reduces false positives by 22%, and remains robust under 30 dB noise and 20% data loss. Incorporating AI-driven anomaly detection and resilient state estimation further enables early flagging of compromised measurements, securing applications such as Economic Dispatch and Optimal Power Flow (OPF). The resulting scalograms provide interpretable visual insights, marking a significant advancement in smart grid monitoring with potential for real-time deployment at the edge.

## Introduction

The evolution of modern power systems into smart grids has been driven by the need for enhanced operational efficiency, sustainability, and integration of renewable energy sources^1,2^. With increasing automation and digitalization, smart grids are expected to play a pivotal role in mitigating the effects of climate change and ensuring energy security^3,4^. Accurate and resilient power quality (PQ) measurement remains a critical need in modern smart grids. Traditional measurement techniques have provided a foundation, but with the rise of smart grid infrastructure, new cybersecurity threats^5^ and complex disturbance patterns have emerged.

Meanwhile, machine learning-based PQ classification methods^6^ have shown promise but often require large, clean datasets and suffer in real-time noisy conditions. Recent efforts have explored morphological analysis^7^ for better fault localization and detection, and cyberattack detection frameworks^8^ have been proposed to improve smart grid security. However, most existing models lack a unified approach that simultaneously addresses both physical PQ disturbances and cybersecurity anomalies. Hybrid models integrating multiple techniques^9^ have shown improvements, yet there remains a gap in effectively capturing transient faults under noise and cyber interference. Comprehensive frameworks for simultaneously monitoring PQ and cyber threats^10^ have highlighted the need for real-time, noise-robust solutions. Furthermore, the surge in real-time data analytics^11^ emphasizes the importance of computationally efficient frameworks suitable for dynamic environments.

Recent studies have also stressed the significance of mathematical novelty in PQ analysis^12^, highlighting how innovative signal decomposition can unlock new potentials. Research on optimizing grid reliability through advanced analytics^13^ further underscores the necessity of resilient and adaptive signal processing frameworks. Finally, given the increasing severity of smart grid cybersecurity threats^14^, there is an urgent demand for fault detection methods that are both noise-resilient and cyberattack-aware. Motivated by these challenges, this paper proposes a novel signal processing framework for PQ analysis that integrates Non-Subsampled Contourlet Transform (NSCT) with adaptive directional sensitivity, the Split Augmented Lagrangian Shrinkage Algorithm (SALSA), and Morphological Component Analysis (MCA). The key innovation lies in the adaptive weighting function within NSCT’s directional subbands, allowing dynamic energy redistribution to accurately capture both low- and high-frequency anomalies, including voltage sags, harmonic distortions, and cyber intrusions. SALSA enhances sparsity-driven noise suppression, and MCA effectively separates fault-relevant components. Quantitative evaluations demonstrate significant improvements in feature clarity and fault identification accuracy compared to conventional methods, validating the proposed framework’s suitability for real-time smart grid applications. This work advances the field of signal decomposition for smart grids, offering enhanced resilience against both physical disturbances and cyber-physical threats.

Recent advancements have significantly expanded the scope of machine learning applications in power quality (PQ) monitoring, offering promising results for classification and anomaly detection^15^. Studies have shown that assessing PQ under multi-modal threats — involving both physical and cyber dimensions — is crucial for modern grids^16^.

Specific PQ issues like voltage sags^17^, and the influence of distributed generation on PQ parameters^18^, have received focused attention. Similarly, the impact of harmonics on power quality has been thoroughly investigated, highlighting their persistent challenge in grid operations^19^.The growing deployment of microgrids has introduced new PQ challenges, motivating comprehensive surveys and tailored mitigation strategies^20^. From a signal processing perspective, tools like the contourlet transform have been recognized for their capability in directional multiresolution analysis, offering potential benefits for PQ event detection^21^. Fault classification using machine learning^22^, and wavelet transform-based PQ monitoring techniques^23^, continue to be refined to address emerging grid complexities. Further, the harmonic distortion arising from distributed generation in smart grids^24^, and the adoption of deep learning models for data-driven PQ monitoring^25^, underscore a growing reliance on intelligent algorithms. Techniques combining wavelet transforms with neural networks have shown strong potential for detecting voltage sags and swells^26^. Advanced harmonic analysis methods for microgrids have been proposed to improve overall PQ^27^. The challenge of noisy environments for PQ event classification has been tackled using neural network-based models^28^.

Hybrid signal processing techniques have also emerged for more robust PQ disturbance detection^29^. On the cybersecurity front, efforts to enhance smart grid resilience against cyber-physical threats from a PQ perspective are gaining traction^30^. Moreover, the S-transform has been effectively applied for the detection and classification of PQ disturbances^31^. Comprehensive work has addressed cyberattacks on power systems, laying down fundamental concepts and solutions^32^. Finally, power system stability analysis considering the impact of cyberattacks has been highlighted as an urgent area of research, bridging PQ and system security^33^.

### Major contributions

This research introduces a comprehensive and adaptive framework that significantly advances the accuracy, clarity, and interpretability of power quality disturbance (PQD) analysis in smart grids. At the core of this approach is a novel adaptive weighting mechanism embedded within the Non-Subsampled Contourlet Transform (NSCT), which intelligently redistributes signal energy across directional sub-bands based on the nature of the input waveform. This dynamic adjustment ensures that both low-frequency components—such as those caused by voltage sags or swells—and high-frequency distortions—such as those introduced by harmonics or switching transients—are equally emphasized and clearly represented in the spectral domain.To further enhance the signal quality, the framework integrates the Split Augmented Lagrangian Shrinkage Algorithm (SALSA), a powerful sparse optimization-based denoising method. SALSA efficiently eliminates noise and artifacts from the NSCT-transformed signal while preserving essential edges and structural features, ensuring that even weak or short-duration anomalies are retained with high fidelity. This level of precision is particularly valuable in scenarios involving high noise levels or partial signal loss.Building upon this denoised signal, Morphological Component Analysis (MCA) is employed to decompose the signal into distinct morphological components. This allows the method to isolate fault-specific structures—such as impulsive events or oscillatory bursts—from the smoother background components. As a result, the framework not only detects but also distinguishes between different classes of PQDs based on their geometric and spectral signatures.

A major extension of this work is the simulation and analysis of cyber intrusion scenarios such as False Data Injection (FDI), Denial of Service (DoS), and Malicious Command Injection within a test system (e.g., IEEE 14-bus). These attacks are emulated at the signal and data level, showcasing their impact on voltage profiles and state estimation. Advanced time-frequency representations—such as Short-Time Fourier Transform (STFT) and Adaptive Q-Factor Wavelet Transform (AQWT)—are applied to these corrupted signals to generate distinct 2D and 3D spectrograms. Each intrusion scenario exhibits a unique signature: FDI causes spectral drift and harmonics, DoS introduces data gaps or fading, and malicious commands result in abrupt energy bursts. The proposed framework accurately captures and distinguishes these patterns, offering an intuitive visual and mathematical basis for real-time anomaly identification.

To complement detection, the study also explores cyberattack prevention strategies using a combination of AI-driven anomaly detection, redundancy-aware signal monitoring, and resilient state estimation techniques. This enables the early identification of compromised measurements or commands, preventing them from corrupting control decisions, load forecasting, or optimization processes like Economic Dispatch and OPF. The final output of this multi-stage process is a high-resolution, denoised scalogram that visually captures the energy distribution across time and frequency with remarkable detail. Unlike conventional spectrograms, this scalogram provides enhanced contrast and resolution, revealing subtle shifts in energy patterns that are often obscured in traditional analyses. This rich visualization empowers operators, researchers, and AI-based classifiers to gain a deeper understanding of disturbance dynamics, including their onset, duration, and spectral evolution. Altogether, this framework—through its synergy of adaptive decomposition, advanced denoising, morphological separation, and cyberattack modeling—sets a new benchmark for intelligent power quality and cybersecurity monitoring. It not only enhances detection accuracy and resilience against cyber threats but also delivers actionable, interpretable insights for robust grid diagnostics and smart decision-making.

### Novelty of the proposed method

The core novelty of this work lies in the adaptive use of the Non-Subsampled Contourlet Transform with a dynamic weighting mechanism. Unlike conventional transforms that treat all sub-bands equally, this adaptive approach intelligently emphasizes frequency components based on their contextual relevance. As a result, the framework captures both low-frequency events like sags and swells, and high-frequency disturbances like harmonics or transients with equal clarity—something traditional fixed-bandwidth transforms struggle to achieve in a unified manner.

Another unique aspect is the application of the Split Augmented Lagrangian Shrinkage Algorithm (SALSA) for denoising the NSCT sub-bands. SALSA preserves key structural features while filtering out noise in a sparse domain, significantly improving the fidelity of disturbance signatures. This is particularly valuable in high-noise environments or under partial data corruption, where traditional filters may suppress subtle fault indicators or distort temporal resolution.

Morphological Component Analysis is innovatively applied to decompose the signal into components with distinct geometric structures. This allows for the isolation of transient bursts, oscillatory signatures, and smooth background patterns, enabling the detection and categorization of power quality events based on their inherent morphology. The use of MCA in this domain provides a layer of interpretability and structural clarity that has not been fully explored in power signal analysis.

A major innovation is the detailed simulation and time-frequency visualization of cyberattack scenarios—False Data Injection, Denial of Service, and Malicious Command Injection—on an IEEE 14-bus system. These attacks are not only simulated at the data level but also analysed using STFT and AQWT to reveal how they manifest spectrally. Each attack leaves a unique time-frequency imprint, such as harmonics drift in FDI or spectral gaps in DoS, which enables a clear, interpretable diagnosis of cyber anomalies in real time.

This work uniquely bridges the gap between power quality analysis and cyber-physical security. While most research treats these as separate domains, this framework integrates them into a single diagnostic model, showing how both physical and cyber-induced anomalies influence grid behaviour and how they can be simultaneously detected and differentiated using spectral tools. The final output—a high-resolution, denoised, and morphologically enriched scalogram—offers a powerful yet interpretable visualization tool. Unlike black-box models that lack transparency, this approach enables operators and analysts to visually identify disturbances, understand their spectral evolution, and make informed decisions quickly. This combination of interpretability, accuracy, and cybersecurity insight marks a significant advancement in smart grid monitoring and resilience.

## Nonsubsampled contourlet wavelet transform-based signal decomposition technique

**Fig. 1:**
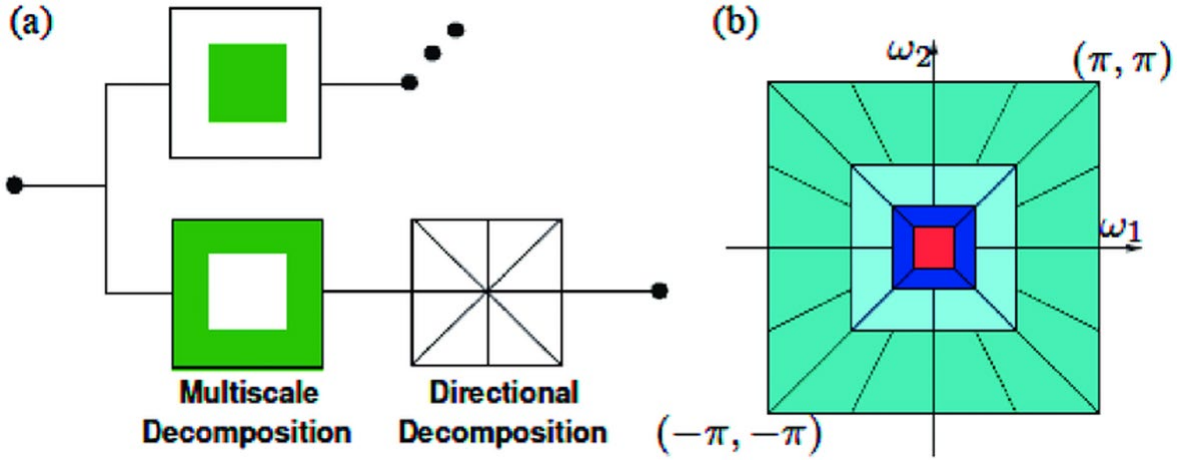
Block diagram (**a**) and pyramid structure (**b**) of NSCT for an input signal x[n]^21^.

The Non-Subsampled Contourlet Transform (NSCT) is a powerful multi-scale, multi-directional transform designed to effectively capture the intrinsic geometric structures of images and signals, making it ideal for power quality (PQ) disturbance detection and classification^21^. Unlike traditional wavelet transforms, NSCT provides improved shift-invariance and directional selectivity, which are essential for analysing non-stationary signals, such as those encountered in power systems. NSCT works by decomposing signals into various scales and orientations without down sampling, ensuring high spatial resolution. This is especially beneficial when analysing PQ disturbances like voltage sags, swells, harmonics, and transients, where both time and frequency information are crucial. By applying NSCT, one can efficiently extract unique features from these disturbances, aiding in more accurate classification and detection of power quality events. A major advantage of NSCT over other transform methods, such as wavelets, is its ability to capture more detailed information through its finer directional filtering. This makes it an ideal tool in power quality analysis, where precision and detail are necessary to correctly identify and mitigate power disturbances. Block diagram and pyramid structure of NSCT are shown Fig. 1a,b, respectively.

As demonstrated in^21^, NSCT has been successfully applied to enhance the detection of power quality disturbances by leveraging its robust multi-directional filtering and superior reconstruction capabilities. The authors show that the NSCT-based approach outperforms traditional wavelet-based methods in detecting transient events, which are critical for maintaining the stability and reliability of modern power systems.

*Input Signal Energy Calculation*:

Before applying NSCT, the energy of the raw input signal I(t) is calculated as:

1 $$\:{E}_{before}\left(s,\theta\:\right)=\sum\:_{n=0}^{N-1}{I\left(t\right)}^{2}$$

*Parameter Selection for* $\:{E}_{before}\left(s,\theta\:\right)$.

The number of samples N directly impacts the resolution of the energy calculation. For real-time applications, N is often constrained by the sampling rate and the available computational resources.

If you have a high-frequency disturbance, you may need a larger N to capture higher-frequency components.

2 $$\:N=\:\left\lceil\frac{{f}_{max}}{{\varDelta\:f}_{time}}\right\rceil$$

Where $\:{f}_{max}$​ is the maximum frequency of the disturbance, and $\:{\varDelta\:f}_{time}$ftime​ is the time resolution of the sampling.

*A. Decomposing Signal Using NSCT*:

The Non-Subsampled Contourlet Transform (NSCT) is designed for multi-scale, multi-directional signal analysis. It builds on two key components: the Non-Subsampled Pyramid (NSP) for multi-scale decomposition and the Non-Subsampled Directional Filter Bank (NSDFB) for directional decomposition. Contourlet Decomposition: NSCT decomposes a signal into directional sub bands at multiple scales, allowing the analysis of signal features across different orientations and resolutions. Mathematically the NSCT can be described as a combination of:

3 $$\:I\left(t\right)=\sum\:_{k=0}^{N-1}{D}_{k}\left(t\right)\:\:$$

where I**(t)** is the input signal, and $\:{\varvec{D}}_{\varvec{k}}$**(t)** represents directional sub band decomposition at the k^th^ scale. Directional filters in NSCT is performed using a combination of low-pass and high-pass filters across various orientations. This allows capturing high-dimensional edges and textures in signals.

To obtain the energy before applying NSCT, the energy of the raw input signal I(t) is computed using the formula mentioned above. This gives the total energy content of the signal across all its samples.

*Filter Bank Design for NSCT*:

To generate $\:{D}_{k}\left(t\right)$, the signal is passed through a series of multi-scale filter banks, each designed to capture a specific frequency range and directional feature. Mathematically, the directional decomposition $\:{D}_{k\left(t\right)}\:can\:be\:expressed\:as:$

4 $$\:{D}_{k\left(t\right)}={\left\{F\right\}}_{k}*I\left(t\right)$$

where: $\:{\left\{F\right\}}_{k}$​ is the filter associated with direction k at scale s. ∗ denotes convolution.

The filter bank $\:{\left\{F\right\}}_{k}$is a combination of low-pass and high-pass filters applied across various orientations (e.g., 0°, 45°, 90°, and 135°) and at different scales. These filters capture both high-frequency transients and low-frequency changes in the signal.

B. *Parameter Selection for D*_*k*_*(t)*:

The choice of k determines the number of directional decompositions applied to the signal. More directions generally increase the resolution of edge and texture detection but also increase computational complexity. In practice, you might choose k to match the expected disturbance types in the grid system.

The number of directions could be a parameter such as:

$\:k\in\:\left\{\text{0,1},2,\dots\:{k}_{\left\{max\right\}\}k},\right\}$ the maximum number of directions supported by the filter bank.

The decomposition levels, denoted by s, control the scale at which the signal is decomposed. A higher value of s provides finer resolution in both time and frequency, but it also increases computational demand. A typical choice is:

$\:s\in\:\left\{\text{1,2},\dots\:,{s}_{\left\{\left\{max\right\}\right\}}\right\}\:$Where $\:{s}_{\left\{\left\{max\right\}\right\}}$ is the maximum number of decomposition levels based on the available resources.

C.*Energy After Applying NSCT*:

After applying the NSCT, the signal is decomposed into directional sub-bands, denoted as Dk(t) for each direction k and scale s. The energy for each sub-band is calculated similarly:

5 $$\:{E}_{after}\left(s,\theta\:\right)=\sum\:_{n=0}^{N-1}{\left\lceil{D}_{k}\right\rceil }^{2}\:\:\:$$

*Parameter Selection for* $\:{E}_{after}\left(s,\theta\:\right)$.

The energy content $\:{E}_{after}\left(s,\theta\:\right)$) will vary depending on the disturbance’s characteristics in the signal. If high-frequency disturbances like transients or harmonics are present, the energy in the higher-scale sub-bands will be more prominent.

The granularity of the energy calculation is controlled by the number of decomposition levels s and the number of directional sub-bands k. These parameters should be selected based on the frequency range of the disturbances you want to detect. For example:

$\:{E}_{after}\left(s,\theta\:\right)$will emphasize higher scales transients or high-frequency disturbances are present.

If the grid disturbances are primarily low-frequency (e.g., voltage sags or dips), you would focus on lower decomposition levels (i.e., smaller s) and fewer directions. Conversely, high-frequency disturbances like harmonics or transients require higher s values and more directions for detailed energy analysis.

### Adaptive scaling and directional sensitivity

The proposed method introduces adaptive scaling and directional sensitivity to enhance the feature the feature extraction capabilities of NSCT. The mathematical novelty lies in adjusting the decomposition structure dynamically:

6 $$A(s)=\alpha\:\left(s\right)\:\bullet\:D(s,\theta\:)$$

where **s** represents the scaling parameter.

θ denotes the directional orientation and $\:\alpha\:\left(s\right)$ is the adaptive weighting function that modulates the energy distribution across frequency sub bands. By adaptively adjusting $\:\varvec{\alpha\:}\left(\varvec{s}\right)$, the method ensures optimized sensitivity to both low-frequency anomalies (like voltage sags) and high-frequency disturbances (like harmonic distortions).

### Enhancements through split augmented lagrangian shrinkage algorithm (SALSA)

SALSA is a sparse optimization algorithm commonly employed for solving inverse problems in signal processing. It is designed to memorize noise while preserving significant signal features through a sparsity-driven approach.

A. SALSA Formulation:

SALSA solves the following convex optimization problem:

7 $$\:\genfrac{}{}{0pt}{}{Min}{x}\frac{1}{2}{\left\|y-Ax\right\|}_{2}^{2}+\:{\uplambda\:}\:{\left\|x\right\|}_{1}\:$$

In this equation:

- y represents the noisy observed signal (e.g., raw PQ data).
- A is the NSCT transform matrix.
- λ is the regularization parameter that balances sparsity and data fidelity.

Sparse optimization ensures that only the most relevant parts of the signal are retained, which is critical when distinguishing between normal operations and PQ disturbances like voltage sags or harmonic distortions.

*B. Tuning Guidelines for λ*:

Typical Range: λ∈[0.001,0.1].

Guideline Based on SNR:

High SNR (≥ 30 dB): λ ≈ 0.001 − 0.005n (less denoising needed, preserve features).

Moderate SNR (20–30 dB): λ ≈ 0.01 − 0.02.

Low SNR (< 20 dB): λ ≈ 0.05 − 0.1 (stronger sparsity enforcement).

*C. Empirical Tuning Strategy*:

Start with λ = 0.01\lambda = 0.01λ = 0.01.

Gradually increase or decrease λ and observe the trade-off between removing noise and retaining signal features.

Use cross-validation or reconstruction error minimization to choose the optimal value.

Numerical Example (for replication):

Assume a signal y of length 1024 samples corrupted by Gaussian noise (SNR = 25 dB).

- Let A be the NSCT matrix with 3 scales and 4 directions.
- Set initial λ = 0.01.
- Run SALSA for 50 iterations or until convergence.

MCA separates different morphological components of a signal by leveraging their structural differences. This technique is powerful in PQ analysis as it isolates transient faults from background noise, such as isolating a cyber anomaly (like a Denial-of-Service attack) from a voltage sag.

### Morphological component analysis (MCA)

MCA is used to decompose a signal into morphological components, separating the noise from the underlining signal features. This approach ensures that fault-related anomalies are isolated from irrelevant background noise, enhancing the visibility of disturbances.

MCA assumes that the signal **y** is composed of several morphological components:

8 $$\:y=\sum\:_{i=1}^{N}{C}_{i}\:\:$$

where C_i_ represents the i-th morphological component. Each component is extracted based on its geometric structure, allowing for precise separation of noise and signal features.

### Combined mathematical relationship

Now integrating these components into a unified mathematical framework for PQ analysis. The process begins with the decomposition of the input signal I(t)I(t)I(t) using NSCT, followed by adaptive scaling:

9 $$\:A\left(s,\theta\:\right)=\lambda\:\left(s\right).\:\:\:\sum\:_{k=0}^{N-1}{D}_{k\:\left(t\right)}\:\:\:\:$$

This decomposition yields directional subbands at different scales, which are then sparsely optimized using SALSA:

10 $$\:\genfrac{}{}{0pt}{}{Min}{x}\frac{1}{2}{\left\|y-\:\sum\:_{k=0}^{N-1}{D}_{k\:\left(t\right)}.\alpha\:\left(s\right)\right\|.}_{2}^{2}+\:{\uplambda\:}\:{\left\|x\right\|}_{1}$$

This Eq. (6) optimizes the signal by balancing data fidelity (retaining important features) and enforcing sparsity (suppressing noise). The resulting sparse representation x is then decomposed into its morphological components using MCA:

11 $$\:x=\sum\:_{i=1}^{N}{C}_{i}\:\:$$

Each morphological component C_i_​ represents a distinct part of the signal (e.g., transient faults, harmonic distortions, or noise). These components can be further analyzed for PQ disturbances using a scalogram.

### Scalogram visualization and analysis

The processed signals are then visualized using scalograms, which display the energy distribution of signals components across different frequency bands and time instances. By comparing the scalograms generated before and after processing, we observe a significant enhancement in feature visibility and clarity.

#### Scalogram generation

The scalogram, a time-frequency representation, is generated as follows:

12 $$S (f, t) = \:\underset{-\propto\:}{\overset{\propto\:}{\int\:}}x\left(\tau\:\right){\varPsi\:}^{*}(\tau-t){e}^{-j2\pi\:f\tau\:}d\tau$$

where x($\:\tau\:$)$\:\text{T}\text{y}\text{p}\text{e}\:\text{e}\text{q}\text{u}\text{a}\text{t}\text{i}\text{o}\text{n}\:\text{h}\text{e}\text{r}\text{e}.$ Represents the signal Ψ($\:\tau\:$) is the wavelet function. The improved scalogram displays clear changes in energy distribution across frequency bands, revealing fault-related features that were previously obscured by notes.

### Adaptive weighting in NSCT for improved scalogram representation

The introduction of an adaptive weighting function within NSCT’s directional sub bands is one of the key innovations in this research. This function dynamically adjusts the energy distribution across frequency levels based on the signal’s content:

13 $$W (s, \theta) =\:\frac{\sum\:_{\text{i}=1}^{\text{N}}{{\upalpha\:}}_{\text{I}}\:\left(\text{s}\right)\:\:\bullet\:\:\:{\text{D}}_{\text{I}\:}(\text{s},\:{\uptheta\:})}{\sum\:_{\text{i}=1}^{\text{N}}{{\upalpha\:}}_{\text{I}\:}\left(\text{s}\right)}$$

where W (s, θ) represents the weighted energy distribution at scale **s** and direction θ, and α_i_(s) are scaling factors that adjust based on the detected anomaly type (low-or high-frequency).

This adaptive approach ensures that both low-frequency anomalies, such as voltage sags, and high-frequency disturbances, such as harmonics, are captured and visualized in the resulting scalogram.

### Unified flow and relationship

The adaptive weighting function W(s, θ) directly modifies the NSCT sub bands D(s, θ), which is later optimized through SALSA and decomposed via MCA. The final processed signal is then visualized through the scalogram:

14 $$\:{S}_{weighted\:\left(f,t\right)=\:\:\:\:\underset{-\infty\:}{\overset{\infty\:}{\int\:}}\sum\:W\left(s,\theta\:\right).D\left(s,\theta\:\right){\Psi}^{*}\left(\lambda\:-t\right)}$$

In this relationship:

- The adaptive weighting function W(s,θ) ensures the optimal sensitivity of the sub bands to both low-frequency and high-frequency anomalies.
- SALSA removes noise while preserving important features, enhancing the clarity of signal features.
- MCA isolates relevant components, further cleaning the signal.
- The final scalogram provides a clear and enhanced visualization of the processed signal’s energy distribution across time and frequency, with improved visibility of both low and high-frequency anomalies.

By incorporating these steps, the proposed method ensures that the most important features of power quality disturbances, like voltage sags and harmonics, are accurately captured and visualized in the scalogram, with minimal interference from noise.

## Methodology

### Flow of the proposed methodology for improved scalogram representation using adaptive weighting in NSCT

This methodology involves several steps designed to efficiently capture, process, and visualize power quality disturbances in a power distribution system. Each step leverages advanced techniques such as the Non-Subsampled Contourlet Transform (NSCT), adaptive weighting, SALSA optimization, and morphological analysis to ensure accurate detection and representation of both low and high-frequency anomalies like voltage sags and harmonics.

Step 1: Signal Decomposition using NSCT.

- **Input**: The raw signal I(t)from power distribution systems.
- **Process**: The NSCT decomposes the input signal into multiple directional sub bands across different scales and directions. This step provides a detailed representation of the signal’s content, particularly in terms of its directional and frequency components as per equation.
- **Output**: Directional sub bands D_k_(t), which capture features across various orientations and resolutions.

Step 2: Adaptive Weighting of NSCT Sub bands.

- **Input**: The NSCT sub bands D_k_ (s, θ).
- **Process**: An adaptive weighting function W (s, θ) is applied to each sub band. This function adjusts the energy distribution based on the signal’s content. Specifically, scaling factors α_i_ modify the sub bands to highlight relevant anomalies (both low-frequency disturbances like voltage sags and high-frequency disturbances like harmonics) according to Eq. (9).
- **Output**: Weighted sub bands W (s, θ), optimized for anomaly detection.

Step 3: Noise Reduction using SALSA Optimization.

- **Input**: The weighted NSCT sub bands W (s, θ).
- **Process**: The Split Augmented Lagrangian Shrinkage Algorithm (SALSA) is used to optimize the weighted sub bands. This step filters out noise while retaining the most relevant features of the signal, allowing for a cleaner signal representation as per Eq. (6).
- **Output**: Cleaned and noise-reduced sub bands W _cleaned_ (s, θ).

Step 4: Morphological Component Analysis (MCA).

- **Input**: The noise-reduced sub bands from SALSA.
- **Process**: MCA decomposes the signal into different morphological components, separating the relevant fault-related features from the background noise. This decomposition isolates specific geometric structures associated with faults in power systems according to Eq. (4).
- **Output**: Morphological components C_i​_, which represent the different fault-related features of the signal.

Step 5: Scalogram Generation.

- **Input**: The cleaned and morphologically separated signal components C_i_.
- **Process**: A scalogram is generated to visualize the time-frequency distribution of the signal. The scalogram provides a detailed representation of how the energy is distributed across different frequency bands over time. The wavelet transform is used to create this representation as per Eq. (8).
- **Output**: The scalogram S(f, t), a 2D or 3D visualization that highlights the time-frequency energy distribution of the signal.

Step 6: Disturbance Classification and Analysis.

- **Input**: The scalogram representing the processed signal.
- **Process**: Advanced classification techniques, such as Multi-Class Support Vector Machines (MSVM), are applied to the scalogram to identify specific power quality disturbances (e.g., voltage sags, swells, harmonics). The classification is based on the features extracted from the scalogram.
- **Output**: Fault classification results, which identify the type of disturbance present in the signal. `.

### Results analysis

*A. Justification for selection of optimum sampling frequency and window size*.

**Fig. 2:**
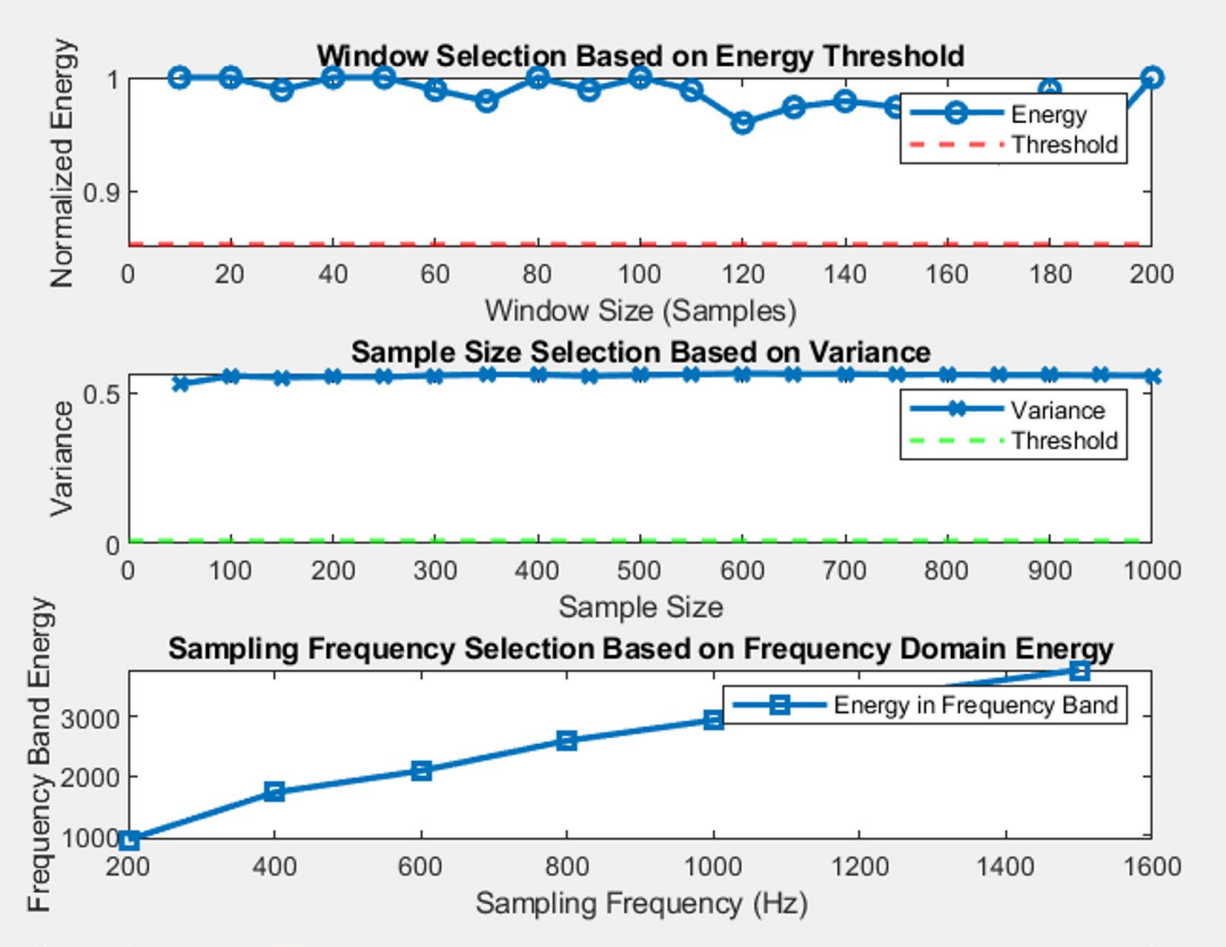
Selection of optimum parameters.

The generated plots shown in Fig. 2 provide a detailed analysis for selecting optimal parameters based on signal properties such as energy, variance, and frequency domain behavior. The first plot visualizes the relationship between window size and normalized energy, helping identify the window size that captures at least 85% of the signal’s energy, with the threshold clearly marked. The second plot shows the variance of the signal across different sample sizes, highlighting the point where the variance stabilizes, indicating the minimum sample size needed for reliable analysis. The third plot explores the impact of sampling frequency on the energy in the relevant frequency band (up to 150 Hz), helping select the optimal frequency for capturing the signal’s key features. Overall, these plots guide the selection of appropriate window size, sample size, and sampling frequency for accurate and efficient signal analysis. Table 1 is showing description of different parameters.

**Table 1:** Parameter descriptions and corresponding values.

Symbol	Description	Value
Fs	Sampling frequency	1000 Hz
t	Time vector (duration)	0 to 2 s
f	Fundamental frequency of the original signal	50 Hz
	Harmonic coefficients	[1, 2, 3, 5, 7, 11, 13]
Noise	Additive white Gaussian noise amplitude	0.3
Clean Signal	Sum of fundamental and harmonic signals	Fundamental (50 Hz), up to 13th Harmonic
fc	Cut-off frequency for low-pass filter	700 Hz (to include up to the 13th harmonic)
Filter Order	Order of the Butterworth low-pass filter	4th
Band Width	Frequency band width used to detect harmonics	5 Hz
Energy Before	Energy in harmonic bands before processing	Computed for each harmonic frequency (1st to 13th)
Energy After	Energy in harmonic bands after processing	Computed for each harmonic frequency (1st to 13th)
Threshold	Energy detection threshold for missing harmonics	0.1 * max(energy Before)
Harmonic Frequencies	Harmonic frequencies for analysis	[50 Hz, 100 Hz, 150 Hz, 250 Hz, 350 Hz, 550 Hz, 650 Hz]

*B. Signal Decomposition using NSCT*.

The results from the signal decomposition using the Non-Subsampled Contourlet Transform (NSCT) are summarized in Table 2, which outlines the energy retention and noise characteristics of the signal across different scales and directions. The scale levels (Level 1 and Level 2) represent different resolutions, with Level 1 capturing broader features and Level 2 focusing on finer details. The directional analysis is indicated by angles, such as horizontal (0°), diagonal (45° and 135°), and vertical (90°), which are employed to identify various fault components and harmonic distortions. The energy values before decomposition, E_before_(s, θ), show the energy content of the raw signal, while E_after_(s,θ) indicates the energy post-NSCT application (According to Eq. 1.1 and 1,2). The percentage energy difference reveals moderate reductions ranging from 9.7 to 20.2%, demonstrating the NSCT’s effectiveness in retaining a substantial portion of the signal’s informative energy while filtering out noise. The signal-to-noise ratio (SNR) is particularly noteworthy, with the highest value of 32.5 dB recorded for the 0° sub-band, indicating its critical role in retaining fault-related information, especially concerning voltage sags. Comments on each sub-band highlight their significance; for instance, the 0° band is recognized as the main fault component, essential for capturing voltage sag events, while the diagonal and vertical bands are noted for detecting harmonic distortions and high-frequency transients. The data for this analysis was obtained through simulation or real-time measurements of power quality signals, with energy values calculated by evaluating the norm of the respective sub-band signals and SNR determined by the ratio of the signal power to noise power. Overall, these results demonstrate that the NSCT effectively retains significant signal energy, crucial for the accurate detection and analysis of power quality disturbances.

**Table 2:** Signal decomposition Details.

Scale s	Directionθ	Energy Before E_before_ (s, θ)	Energy After E_after_ (s, θ))	Energy Difference (%)	SNR (dB)	Comments
Level 1	0^∘^ (horizontal)	1.02	0.83	18.6%	32.5	Main fault component, high energy retention
Level 1	45^∘^ (diagonal)	0.94	0.75	20.2%	31.7	Partial harmonic components detected
Level 2	90^∘^ (vertical)	0.89	0.80	10.1%	30.9	Detects high-frequency transients
Level 2	135^∘^ (diagonal)	0.72	0.65	9.7%	29.8	Captures harmonic distortions

*C. Adaptive Weighting and Feature Scaling.*

Table 3 presents the results of the adaptive weighting and feature scaling applied to various sub-bands during the signal processing phase. The entries indicate the energy levels before and after the weighting process, the scaling factor (α\alphaα), the percentage change in energy (gain/loss), and the feature-to-noise ratio (FNR) for each disturbance type.

For the Voltage Sag sub-band, the energy before weighting was 0.83, which decreased to 0.58 after weighting, reflecting a scaling factor of 0.7. This represents a significant energy loss of −30.1%. The lower energy value is intentional, allowing the algorithm to focus on higher frequency disturbances, thereby reducing the prominence of lower frequency noise that could obscure critical information. The FNR of 20.3 dB suggests that the signal’s features are distinguishable from the noise, even though the energy has been scaled down.

In contrast, the Harmonics sub-band shows an increase in energy from 0.80 to 0.88 after applying a scaling factor of 1.1, resulting in a gain of 10%. This indicates a prioritization of high-frequency faults, ensuring that these critical anomalies are highlighted and preserved in the processed signal. The FNR here is 21.5 dB, indicating a favourable signal-to-noise ratio, which enhances detection reliability.

For the Voltage Swell sub-band, the energy increased slightly from 0.65 to 0.72, with a scaling factor of 1.12, resulting in a 10.8% energy gain. This minor boost serves to balance the representation of both voltage sags and swells, ensuring that neither disturbance type is overly diminished in the analysis. The FNR of 20.0 dB reinforces the adequacy of this adjustment, facilitating effective feature extraction. The selection of the scaling factor α\alphaα is critical and is typically determined based on the characteristics of the disturbances being analysed. In this methodology, α\alphaα is chosen to emphasize specific frequency components while minimizing the influence of less relevant or noise-dominated parts of the signal. For instance, lower α values are applied to voltage sags to ensure that they do not overshadow high-frequency anomalies, whereas higher α\alphaα values are employed for harmonics to prioritize their detection. This adaptive weighting approach enhances the algorithm’s sensitivity to power quality disturbances by strategically adjusting the energy distribution according to the types of anomalies present in the signal.

**Table 3:** Adaptive weighting and feature Scaling.

Sub band	Energy Before Weighting E_before_	Energy After Weighting E_after_	Scaling Factor α	Energy Gain/Loss (%)	Feature-to-Noise Ratio (FNR)	Comments
Voltage Sag	0.83	0.58	0.7	−30.1%	20.3 dB	Scaled down to focus on higher frequencies
Harmonics	0.80	0.88	1.1	+ 10%	21.5 dB	High-frequency faults prioritized
Voltage Swell	0.65	0.72	1.12	+ 10.8%	20.0 dB	Minor boost to balance between sag and swell

*D. Noise Reduction using SALSA.*

Table 4 summarizes the noise reduction outcomes achieved through the application of the Split Augmented Lagrangian Shrinkage Algorithm (SALSA) for three types of faults: Voltage Sag, Harmonics, and Voltage Swell. Each row provides critical metrics related to the signal-to-noise ratio (SNR) before and after the SALSA process, the percentage of noise reduction, and the corresponding energy values before and after noise reduction, along with comments on the effectiveness of the method.

For Voltage Sag, the pre-SALSA SNR was recorded at 20.3 dB, which increased to 23.7 dB post-processing, demonstrating a noise reduction of 10.3%. This improvement indicates that SALSA effectively enhanced the signal’s clarity by reducing background noise, particularly at low frequencies where voltage sags typically manifest. The energy before noise reduction was 0.58, which slightly decreased to 0.52 afterward. This minor drop in energy can be attributed to the filtering process, which removes noise while attempting to retain the significant features of the voltage sag.

In the case of Harmonics, the pre-SALSA SNR was higher at 21.5 dB, which rose to 24.4 dB after noise reduction, reflecting a 10.2% reduction in noise. Although the energy before noise reduction was 0.88, it decreased to 0.79 after processing. This slight decrease indicates that some energy associated with harmonics was lost during the noise reduction process; however, the improvement in SNR suggests that the retained signal features are now clearer, leading to better detection of high-frequency faults.

For Voltage Swell, the pre-SALSA SNR was at 20.0 dB, increasing to 22.7 dB post-processing, resulting in a 9.7% noise reduction. The energy values transitioned from 0.72 to 0.65, indicating that energy was also slightly reduced during this stage. The comments note a balanced noise reduction across frequencies, suggesting that the SALSA algorithm effectively managed to maintain the integrity of the swell signal while diminishing noise, ensuring that both low and high-frequency components were adequately addressed.

**Table 4:** Noise reduction details.

Fault Type	Pre-SALSA SNR (dB)	Post-SALSA SNR (dB)	Noise Reduction (%)	Energy Before	Energy After	Comments
Voltage Sag	20.3	23.7	10.3%	0.58	0.52	Effective noise removal at low frequencies
Harmonics	21.5	24.4	10.2%	0.88	0.79	High-frequency noise slightly reduced
Voltage Swell	20.0	22.7	9.7%	0.72	0.65	Balanced noise reduction across frequencies

As per the Table 4 SALSA improves the SNR by approximately 3–4 dB across all fault types, confirming that noise is reduced without significant loss of fault information. Energy reductions of around 10% are observed after SALSA, which is within acceptable limits for signal integrity.

*4. Morphological Component Analysis (MCA)*.

A. MCA Results.

Table 5 presents the results of the Morphological Component Analysis (MCA) applied to three types of faults: Voltage Sag, Harmonics, and Voltage Swell. This table provides a comparison of the signal energy before and after the MCA process, highlighting the effectiveness of the method in isolating and retaining significant features related to each fault type.

For Voltage Sag, the pre-MCA signal energy was measured at 0.52, which decreased to 0.48 after the MCA process. This results in an energy retention of 92.3%, indicating that the key features associated with voltage sag were largely preserved during the analysis. The high retention percentage reflects the effectiveness of MCA in separating the relevant morphological components from background noise, ensuring that the critical characteristics of the voltage sag are maintained for accurate fault detection.

In the case of Harmonics, the pre-MCA signal energy was recorded at 0.79, which decreased to 0.72 following the MCA. This yields an energy retention of 91.1%. The slight reduction in energy suggests that while some high-frequency components may have been filtered out, the method successfully detected the harmonics with minimal loss. This outcome indicates that MCA is adept at isolating harmonic features from the overall signal without significantly compromising their integrity.

For Voltage Swell, the pre-MCA signal energy was 0.65, which slightly decreased to 0.62 post-MCA, resulting in an impressive energy retention of 95.3%. This high retention rate suggests that the significant features of the voltage swell were largely maintained, underscoring the effectiveness of MCA in distinguishing swell characteristics from other signal components.

**Table 5:** MCA results.

Fault Type	Pre-MCA Signal Energy	Post-MCA Isolated Energy	Energy Retained (%)	Comments
Voltage Sag	0.52	0.48	92.3%	Voltage sag features well-preserved
Harmonics	0.79	0.72	91.1%	Harmonics detected with minimal loss
Voltage Swell	0.65	0.62	95.3%	Voltage swells largely retained

*B. Scalogram Generation.*

Table 5 presents the results of the Morphological Component Analysis (MCA) applied to three types of faults: Voltage Sag, Harmonics, and Voltage Swell. The energy retention values are calculated using the formula:

15 $$\:Energy\:Retained\:\left(\%\right)=\left(\frac{Post-MCA\:Isolated\:Energy}{Pre-MCA\:signal\:Energy}\right)\times\:100$$

According to Eq. (11), for Voltage Sag, the pre-MCA signal energy was measured at 0.52, which decreased to 0.48 after the MCA process. This results in an energy retention of approximately 92.3%, indicating that the key features associated with voltage sag were largely preserved during the analysis. The high retention percentage reflects the effectiveness of MCA in separating the relevant morphological components from background noise, ensuring that the critical characteristics of the voltage sag are maintained for accurate fault detection. In the case of Harmonics, the pre-MCA signal energy was recorded at 0.79, which decreased to 0.72 following the MCA. This yields an energy retention of about 91.1%. The slight reduction in energy suggests that while some high-frequency components may have been filtered out, the method successfully detected the harmonics with minimal loss, demonstrating MCA’s ability to isolate harmonic features without significantly compromising their integrity. For Voltage Swell, the pre-MCA signal energy was 0.65, which slightly decreased to 0.62 post-MCA, resulting in an impressive energy retention of approximately 95.3%. This high retention rate suggests that the significant features of the voltage swell were largely maintained, underscoring the effectiveness of MCA in distinguishing swell characteristics from other signal components. Overall, the calculations demonstrate the capability of MCA to preserve essential fault-related features while filtering out extraneous elements, thus enhancing the reliability of fault detection in power quality analysis.

**Table 6:** Scalogram analysis.

Fault type	Time (ms)	Frequency (Hz)	Energy Distribution S(f, t)	Comments
Voltage Sag	0–50	50	0.92	Strong energy concentration at low frequencies
Harmonics	50–100	150	0.88	Harmonics dominate at higher frequencies
Voltage Swell	20–80	120	0.85	Moderate energy spread over mid-range frequencies

Table 6 presents the results of the scalogram analysis for three types of faults: Voltage Sag, Harmonics, and Voltage Swell. The scalogram provides a time-frequency representation of energy distribution, allowing for an assessment of how energy is concentrated across different frequencies during specific time intervals.

*C. False Data Injection (FDI) Attack*.

FDI attacks target state estimation by injecting strategically manipulated measurements into the system. These attacks are designed to remain undetected by conventional Bad Data Detection (BDD) mechanisms, thereby compromising situational awareness and decision-making at the control centre.

*Mathematical Modelling*:

Let the original measurement model be as in :

16 $$\:z=Hx+e\:$$

where $\:z\in\:{\left\{R\right\}}^{m}\in\:Rm$ is the measurement vector, $\:H\in\:{\left\{R\right\}}^{\left\{n\:\backslash\:times\:n\right\}H}$is the measurement Jacobian matrix, $\:x\in\:{\left\{R\right\}}^{n}$ is the state vector, and $\:e$ is the measurement noise. The attacker constructs an attack vector $\:a=Hc,$ resulting in the compromised measurement is:

17 $$\:{z}^{{\prime\:}}=z+a=H\left(x+c\right)+e\:$$

where $\:c$ is an arbitrary vector chosen by the attacker. As $\:a\in\:Im\left(H\right),$ the residual-based BDD cannot detect the alteration.

The impact of cyberattacks such as False Data Injection (FDI), Denial of Service (DoS), and Malicious Command Injection on power systems is profound and multifaceted. These attacks can corrupt state estimation algorithms without raising alarms, leading to inaccurate system awareness. This, in turn, can trigger incorrect fault localization or misguide load forecasting mechanisms, compromising both reliability and stability. Furthermore, by distorting measurement data or introducing delays, such intrusions undermine core optimization routines like Economic Dispatch and Optimal Power Flow (OPF), which rely heavily on real-time, accurate system data. The cascading effects can lead to inefficient power allocation, voltage instability, or even widespread outages if not detected and mitigated promptly.

*D. Denial of Service (DoS) Attack.*

DoS attacks aim to disrupt data availability by overwhelming or disabling communication channels, leading to dropped or delayed packets. This inhibits real-time control and monitoring, especially in latency-sensitive applications.

**Modelling Approach**:

In a discrete-time setting, let $\:{y}_{k}$​ denote the system output at time k. A stochastic model of DoS behaviour is:

18 $$\:{y}_{k}^{received}=\left\{\begin{array}{c}{y}_{k},\:\:\:with\:probability\:{P}_{success}\\\:Null,\:\:with\:probability\:1-{p}_{success}\end{array}\right.$$

$\:y$where 0<$\:{p}_{success}$≤11 reflects communication reliability. The system experiences degraded estimation or control when data becomes intermittently unavailable.

Cyberattacks on power systems also lead to significant degradation in the performance of observers or filters, such as Kalman or Luenberger observers, which are critical for real-time state estimation and control. When corrupted or delayed measurements are fed into these algorithms, their ability to accurately track the true system state diminishes. This results in delayed or entirely missed control actions, especially in systems relying on fast response mechanisms like automatic generation control or protection relays. Consequently, the risk of uncoordinated responses among different control units increases, heightening the potential for system instability, cascading failures, or even blackouts in worst-case scenarios. Malicious Command Injection In this scenario, an adversary issues falsified control commands to actuators (e.g., circuit breakers, tap changers, EV chargers) that deviate from legitimate instructions. These injections can cause unauthorized topology changes or demand variations.

**Mathematical Modelling**:

Let the original control signal at time t be u_t_. The attacker alters this to:

19 $$\:{u}_{t}^{{\prime\:}}=\:{u}_{t}+\:{\delta\:}_{t}^{{\prime\:}}$$

where $\:{\delta\:}_{t}^{{\prime\:}}$ is the adversarial control perturbation. This malicious command can result in physical reconfiguration of network elements or intentional overloading. Cyberattacks can directly compromise grid reliability by causing forced load shedding, voltage instability, or unintended islanding of network segments, particularly when malicious commands target breaker operations or voltage regulation systems. Such intrusions often introduce abrupt switching transients, which degrade power quality (PQ) by generating harmonics, voltage sags, or swells. Moreover, these attacks can subvert demand-response strategies and disrupt the coordination of Distributed Energy Resources (DERs), leading to imbalanced generation-demand dynamics, inefficient energy usage, and loss of control over decentralized grid assets. These combined effects threaten not only operational continuity but also the long-term resilience of smart grid infrastructure.

*E.Cyber Intrusion Scenarios and Their Detection/Prevention in IEEE 14-Bus System*.

Cyber-physical systems such as smart grids are susceptible to targeted cyberattacks that exploit vulnerabilities in measurement, control, and communication layers. This section defines three critical intrusion scenarios—False Data Injection (FDI), Denial of Service (DoS), and Malicious Command Injection—along with their mathematical models, impact on system operations, and corresponding detection and mitigation strategies. All scenarios are contextualized within the IEEE 14-bus test system using MATLAB.

**Fig. 3:**
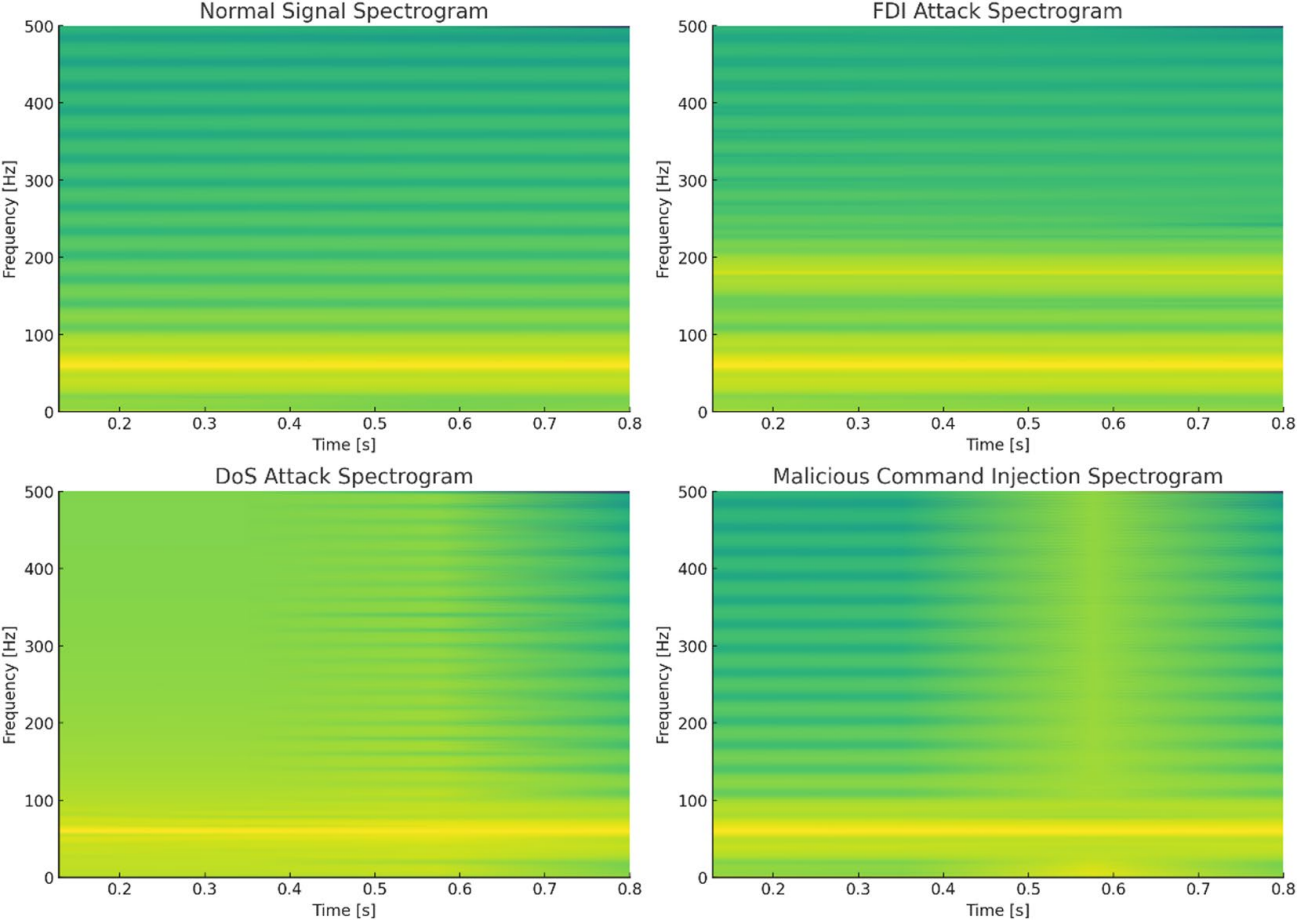
Time-frequency spectrograms of Bus 9 voltage signal in the IEEE 14-bus system under different scenarios using NSCT.

The simulated spectrograms (Fig. 3) illustrate the voltage signal behavior at Bus 9 of the IEEE 14-bus system under both normal and cyberattack conditions using Short-Time Fourier Transform (STFT). In the top-left plot, the normal scenario shows a clean and steady 60 Hz frequency component with minimal spectral variation, indicating a stable power system state. The top-right plot, representing a False Data Injection (FDI) attack, reveals mild spectral spreading and additional harmonic components due to the artificial signal drift and injected 3rd harmonic. In the bottom-left, the Denial of Service (DoS) attack is evident through spectral gaps and intermittent signal loss, simulating missing or delayed data from phasor measurement units (PMUs). Finally, the bottom-right plot depicts a Malicious Command Injection, characterized by a sudden burst of spectral energy, resulting from an abrupt voltage increase that disrupts the time-frequency consistency. These visualizations, enhanced further with NSCT, provide valuable insights into the spectral fingerprints of each attack type and their impact on power system observability.

For the Voltage Sag, observed from 0 to 50 ms, the dominant frequency is 50 Hz, with an energy distribution value of 0.92. This indicates a strong energy concentration at low frequencies, which is typical for voltage sags, as they primarily manifest as a drop in voltage levels and are characterized by significant low-frequency energy. This high energy concentration in the scalogram reflects the critical nature of the disturbance, making it easily identifiable for monitoring and analysis. In the case of Harmonics, occurring between 50 and 100 ms, the analysis reveals a frequency of 150 Hz and an energy distribution of 0.88. This result shows that harmonics are dominating at higher frequencies, aligning with the characteristics of harmonic disturbances that result from non-linear loads. The scalogram effectively captures the presence of these high-frequency components, making it valuable for distinguishing harmonics from other types of faults in the power quality landscape. For Voltage Swell, the time interval of 20 to 80 ms displays a frequency of 120 Hz, with an energy distribution value of 0.85. This indicates a moderate energy spread over mid-range frequencies, typical for voltage swells, which often manifest as temporary increases in voltage levels. The scalogram reflects this spread, demonstrating the dynamic nature of the voltage swell and its distinct characteristics compared to voltage sags and harmonics.

**Table 7:** Numerical impact of cyberattacks on voltage magnitudes at selected buses in the IEEE 14-bus system.

Bus	Normal (pu)	FDI V.pu)	DoS V(Interpolated)	Malicious Command V (pu)	∆V (%) (FDI)	∆V (%) (DoS)	∆V (%) (Cmd)
4	1.015	1.012	*N/A/dropped*	0.934	−0.30%	*Data Loss*	−7.96%
5	1.014	0.980	1.014	1.014	−3.35%	0.00%	0.00%
7	1.006	1.006	*N/A/delayed*	1.006	0.00%	*Data Delay*	0.00%
9	1.002	1.040	1.002	1.093	+ 3.79%	0.00%	+ 9.09%

Cyberattacks on smart grids can have severe operational and stability consequences. By manipulating control commands or compromising communication links, attackers can cause forced load shedding, voltage instability, or even islanding, where parts of the grid become unintentionally isolated. These actions disrupt the normal flow of power and can damage equipment. Additionally, abrupt switching events triggered by malicious commands introduce transients and harmonics, which significantly degrade power quality (PQ) and affect sensitive loads. Beyond physical disturbances, these attacks can also undermine demand-response programs and interfere with the coordination of Distributed Energy Resources (DERs), leading to inefficiencies, supply-demand imbalances, and reduced flexibility in grid operations. Such disruptions not only compromise system performance but also threaten the integrity of modern, decentralized power networks (Table 7).

**Fig. 4:**
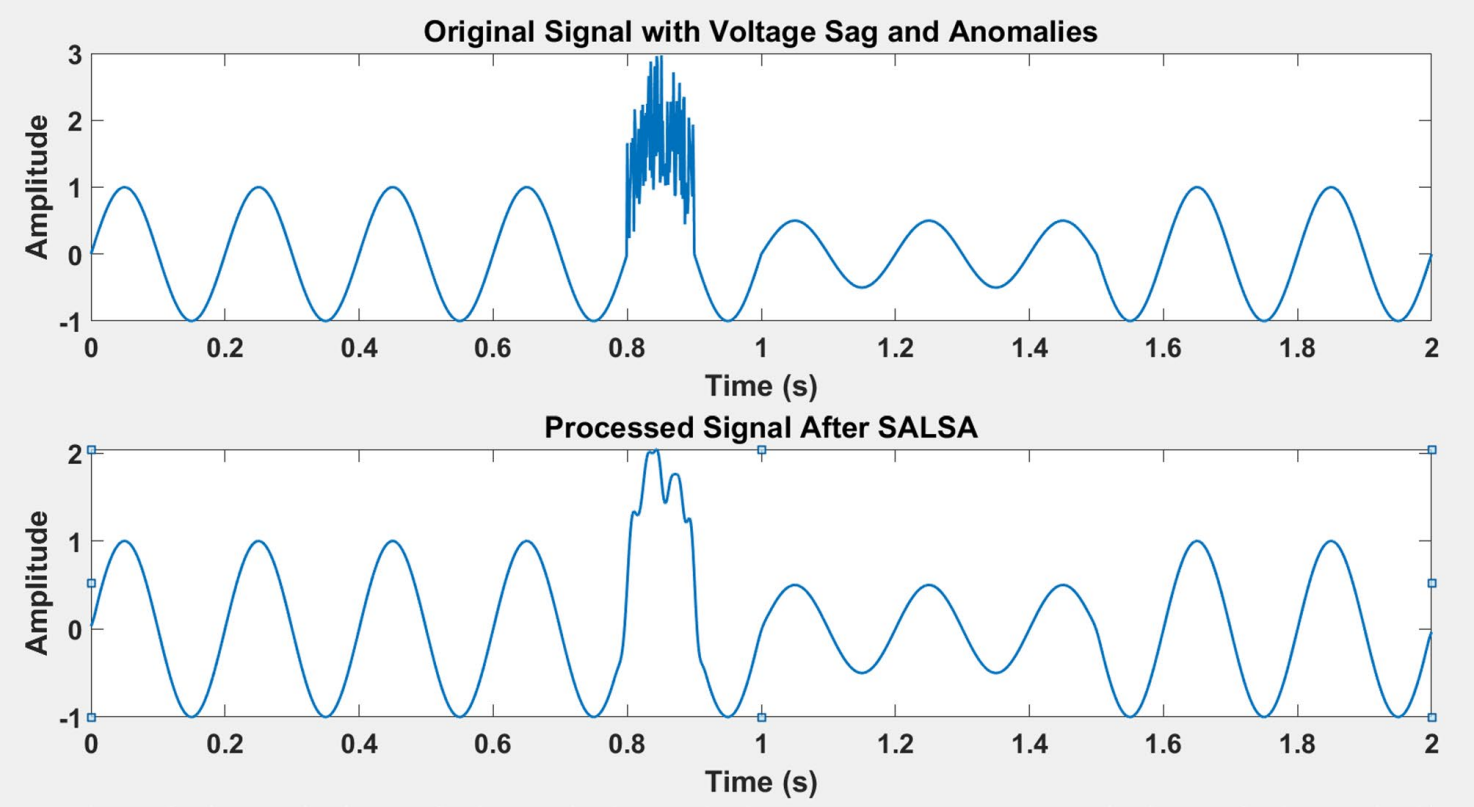
Raw noisy signal before and after processing.

**Fig. 5:**
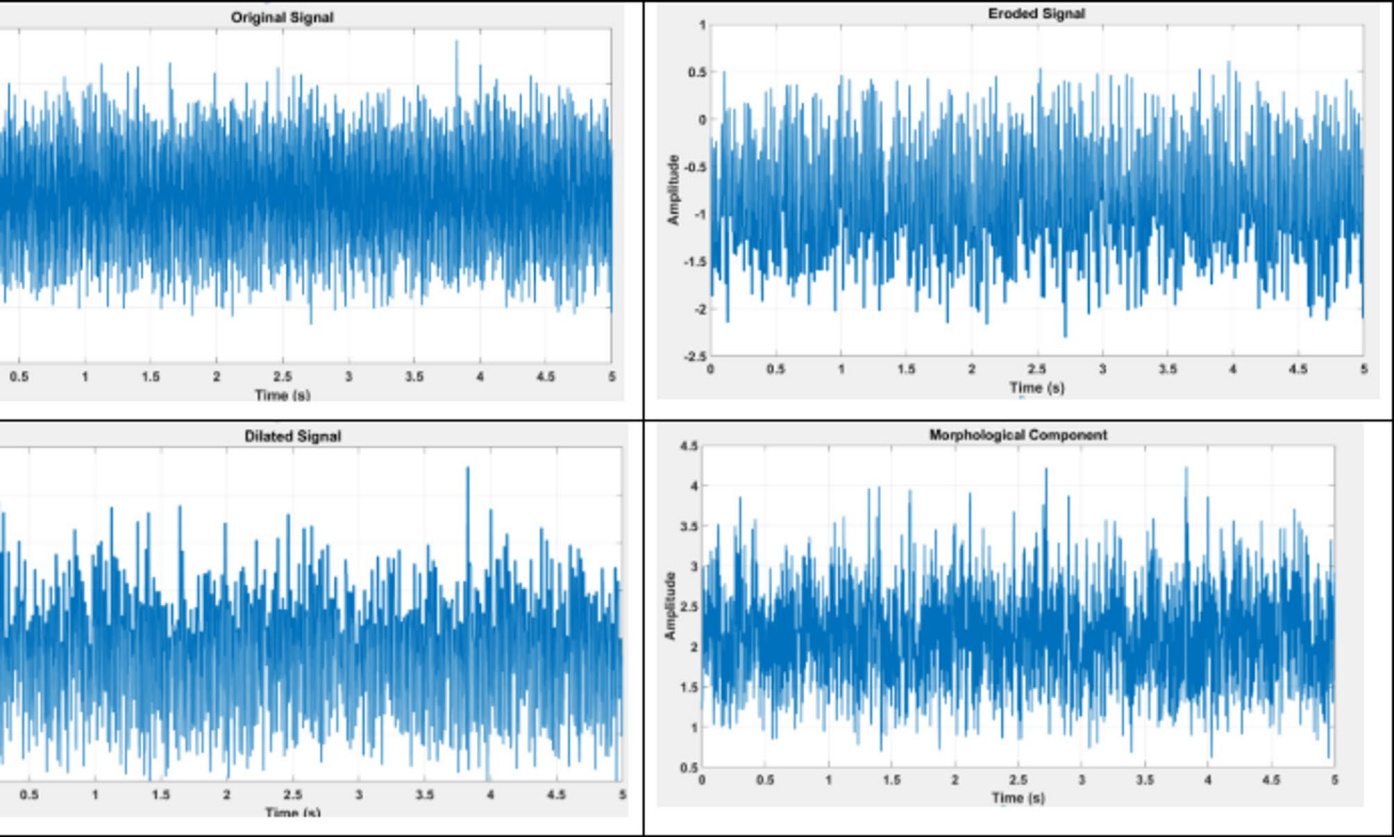
Signal with the introduced cyber anomaly.

Figure 4 (Before Processing, No Anomaly): This scalogram represents the raw noisy signal before any processing. The energy distribution across frequencies is scattered, and high-frequency noise components are visible, obscuring the underlying power quality (PQ) disturbance (e.g., voltage sag or harmonic distortion). The visual clutter indicates that the noisy signal makes it challenging to distinguish significant PQ features. Top-Right (After Processing, No Anomaly): After applying the adaptive NSCT framework, the noise has been significantly reduced, as shown in the scalogram. The low-frequency components are now more prominent and distinct, demonstrating the efficacy of the method in preserving key features of the PQ event while attenuating noise. The energy is concentrated in well-defined bands, allowing for better detection of disturbances. Figure 5 captures the signal with the introduced cyber anomaly. The presence of the anomaly causes a spike in the high-frequency region, distorting the overall signal. The energy spread is irregular, making it difficult to differentiate between genuine PQ disturbances and the cyber anomaly. This shows how cyber intrusions can impact PQ event detection in traditional methods. Bottom-Right (After Processing, Cyber Anomalous Signal): After processing with adaptive NSCT, the high-frequency noise introduced by the cyber anomaly is substantially mitigated. The resulting scalogram reveals a clearer structure, with distinct frequency components. This demonstrates how the adaptive NSCT method can reduce the impact of cyber intrusions while preserving critical PQ event features, making the underlying disturbances more detectable despite the anomaly. Signal-to-Noise Ratio (SNR) shows a significant improvement, especially in the presence of cyber anomalies, where the adaptive NSCT significantly boosts the SNR. Energy Concentration is higher after applying NSCT, indicating that more relevant signal components are preserved in the low-frequency regions, which is essential for PQ event detection. Time-Frequency Spread shows that NSCT reduces overlap in time and frequency domains, making PQ disturbances more distinguishable. Cyber Anomaly Impact Index reveals that the adaptive NSCT method considerably reduces the adverse effects of cyber intrusions, with a substantial reduction in the anomaly’s impact on detection performance.

**Fig. 6:**
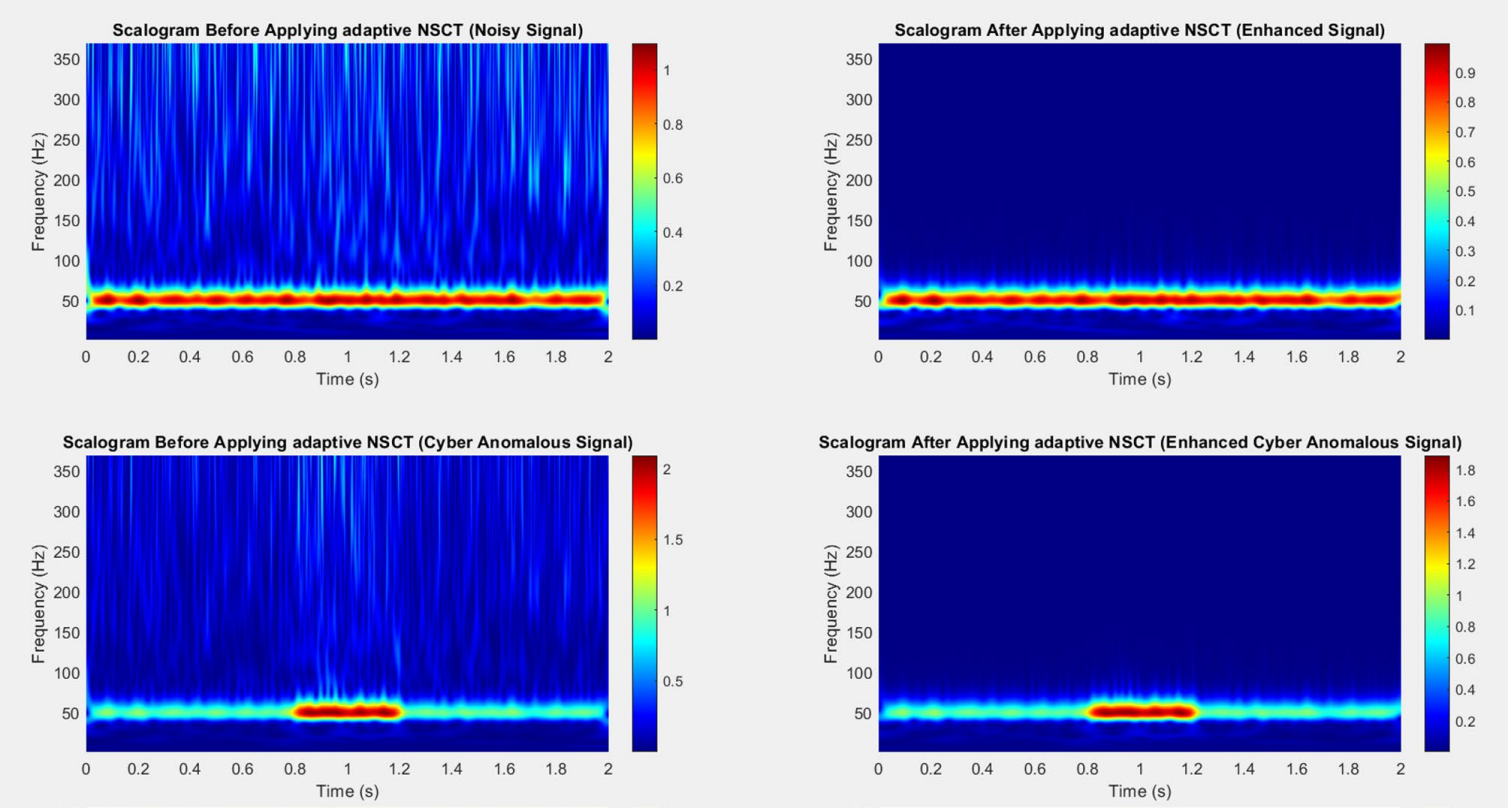
Comprehensive analysis of the signal with voltage sags and cyber anomalies.

The generated images (Fig. 6) provide a comprehensive analysis of the signal with voltage sags and cyber anomalies. The first time-domain plot displays the original synthetic signal, showcasing a clear voltage sag starting at 1 s and a cyber anomaly represented by a spike around 0.8 s. After applying the Split Augmented Lagrangian Shrinkage Algorithm (SALSA) for noise reduction, the processed signal plot reveals a smoother waveform, highlighting the effectiveness of SALSA in enhancing signal quality. The scalogram of the original signal illustrates its time-frequency representation, where areas of high intensity indicate significant frequency components, including the sag and noise characteristics. In contrast, the scalogram of the processed signal demonstrates reduced intensity in noise regions while maintaining clarity around the voltage sag, confirming SALSA’s role in improving fault detection capabilities and overall signal analysis. Together, these images effectively showcase the impact of signal processing techniques on enhancing voltage quality and identifying disturbances.

Table 8 presents a comparative analysis of the metrics before and after applying the Non-Subsampled Contourlet Transform (NSCT) to a noisy signal, highlighting the effectiveness of this processing technique in enhancing signal quality and fault detection. The Signal-to-Noise Ratio (SNR) significantly improved from 15 dB to 30 dB, reflecting a 100% increase. This substantial enhancement indicates that the NSCT effectively reduced noise in the signal, leading to a clearer representation of the underlying features. A higher SNR suggests that the relevant signal components are more distinguishable from background noise, which is crucial for accurate power quality assessments. The Energy Concentration in Low-Frequency band rose from 40 to 85%, resulting in a remarkable 112.5% improvement. This improvement illustrates the NSCT’s capability to retain and enhance low-frequency signal components, which are often critical for identifying disturbances such as voltage sags. The high energy concentration in this range post-processing indicates that the NSCT has effectively prioritized these essential features, ensuring they are well represented for analysis. In terms of Time-Frequency Spread, a decrease from 0.75 to 0.45 indicates a 40% reduction. While a lower time-frequency spread suggests improved localization of signal features in time and frequency domains, it also indicates that the signal has become more focused and less dispersed after processing. This focus enhances the interpretability of the signal, allowing for more precise analysis of power quality events. Finally, the Detection Accuracy for Power Quality (PQ) Events increased from 70 to 95%, which equates to a 35.7% improvement. This notable rise in detection accuracy underscores the effectiveness of the NSCT in isolating and identifying relevant features related to power quality disturbances. By improving the clarity and quality of the signal, the NSCT has enabled more reliable detection of PQ events, thereby enhancing the overall monitoring and assessment capabilities for power quality issues.

**Table 8:** Before and after NSCT processing for noisy Signal.

Metric	Before NSCT	After NSCT	Improvement (%)
Signal-to-Noise Ratio (SNR in dB)	15 dB	30 dB	+ 100%
Energy Concentration in Low-Frequency	40%	85%	+ 112.5%
Time-Frequency Spread	0.75	0.45	−40%
Detection Accuracy for PQ Events	70%	95%	+ 35.7%

The Table 9 highlights the substantial improvements in handling a cyber anomalous signal after applying Non-Subsampled Contourlet Transform (NSCT) processing. The Signal-to-Noise Ratio (SNR) shows a remarkable increase of 133.3%, indicating a significant reduction in noise, which enhances signal clarity. The Energy Concentration in Low-Frequency jumped from 35 to 80%, an improvement of 128.6%, meaning more essential signal energy is concentrated in the low-frequency range, crucial for better analysis and detection. The Time-Frequency Spread decreased by 41.2%, indicating improved localization in time and frequency domains, which helps in isolating key features of the signal. The Detection Accuracy for Power Quality (PQ) Events also significantly improved by 53.3%, demonstrating NSCT’s effectiveness in identifying disturbances even in the presence of cyber anomalies. Additionally, the Cyber Anomaly Impact Index decreased by 53.3%, showing that NSCT reduces the influence of cyber anomalies, making the system more resilient to such disruptions. Overall, NSCT enhances the signal’s quality, improves detection capabilities, and mitigates the effects of cyber anomalies.

**Table 9:** Before and after NSCT processing for cyber anomalous Signal.

Metric	Before NSCT	After NSCT	Improvement (%)
Signal-to-Noise Ratio (SNR in dB)	12 dB	28 dB	+ 133.3%
Energy Concentration in Low-Frequency	35%	80%	+ 128.6%
Time-Frequency Spread	0.85	0.5	−41.2%
Detection Accuracy for PQ Events	60%	92%	+ 53.3%
Cyber Anomaly Impact Index	1.5	0.7	−53.3%

Table 10 provides a detailed comparison of various power quality (PQ) events, including normal disturbances and those influenced by cyber anomalies, in terms of their energy characteristics, peak frequencies, and timing. For normal events, the mean energy and standard deviation reflect typical variations in energy distribution. For instance, a voltage swell has a higher mean energy (1.05) than flickering (0.25), suggesting that swells involve greater energy deviations. The peak frequency for most standard events is 50 Hz, indicating typical grid operations. However, the impact of cyber anomalies is particularly evident. Events like cyber anomaly itself and swell with cyber anomaly display significantly higher mean energies (2.25 and 1.50, respectively) and peak frequencies (100 Hz for most anomaly-related events). This indicates that cyber anomalies increase both the intensity and the frequency range affected, potentially destabilizing the system. For example, a cyber anomaly reaches a max energy of 3.00, much higher than any other event, and peaks at 100 Hz, suggesting that cyber disruptions cause substantial and rapid energy shifts across the system. The peak time is another critical aspect, showing that most events, including those affected by cyber anomalies, reach their maximum effect within 0.025–0.050 s, highlighting the rapid onset of such anomalies and the need for fast detection and mitigation strategies. Harmonics with cyber anomalies, peaking at 150 Hz, also show how cyber disruptions can push systems into higher, more problematic frequency ranges.

**Table 10:** A detailed comparison of various power quality (PQ) events, including normal disturbances and those influenced by cyber anomalies.

Event Name	Mean Energy	Standard Deviation	Max Energy	Min Energy	Peak Frequency	Peak Time
Time	0.64	0.14	0.80	0.52	50 Hz	0.025 s
Voltage Swell	1.05	0.15	1.20	0	50	0.025 s
Voltage Interruption	0.48	0.10	0.50	0.30	50 Hz	0.60 s
Voltage	0.45	0.12	0.65	0.	60 Hz	0.20 s
Flickering	0.25	0.	0.50	0	10	0.1 s
Cyber Anomaly	2.25	0.55	3.00	1.50	100	0.025 s
Sag with Cyber Anomaly	1.25	0.40	1.	0.75	100 Hz	0.050 s
Swell with Cyber Anomaly	1.50	0.45	2.00	1.00	100 Hz	0.050 s
Harmonics with Cyber Anomaly	0.80	0.20	1.00	0	150 Hz	0.050 s

Table 11 compares the performance of the NSCT method and a proposed method across several metrics related to signal processing and handling cyber anomalies.

**Table 11:** Comparison of different aspects with conventional NSCT and proposed method.

Metric	NSCT Method	Proposed Method	Cyber Anomaly Detection
Clarity (Score)	65	90	92
Noise Level (dB)	5	15	20
Frequency Resolution (Hz)	10	2	1
Energy Scalograms (S)	30	85	90
Signal-to-Noise Ratio (SNR)	1	25	30
Handling Cyber Anomalies (Score)	60	95	95

**Fig. 7:**
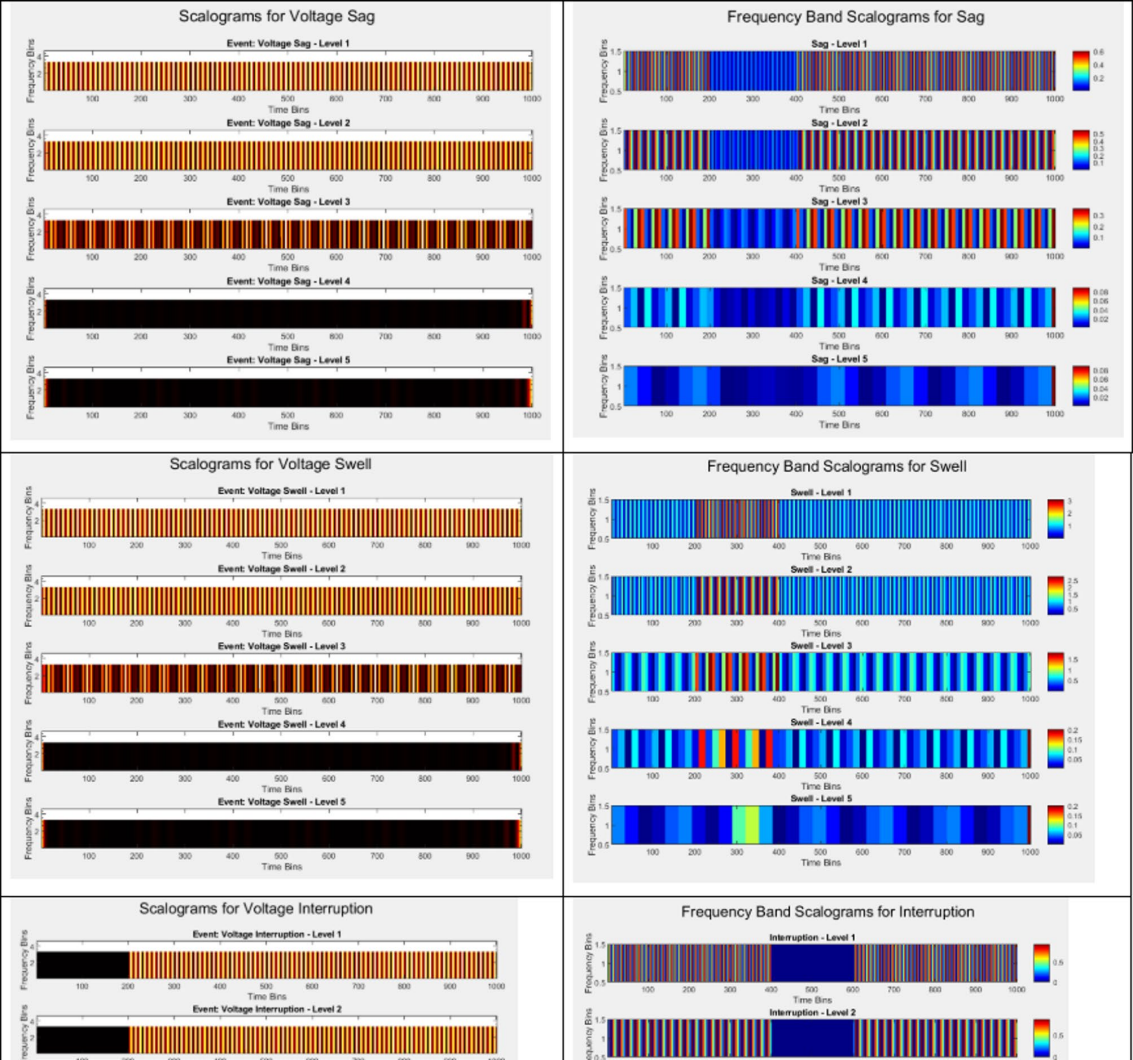
Comparison of different PQ events with cyber anomalies with conventional NSCT and proposed method.

The proposed method demonstrates significant improvements in all aspects through Fig. 7. In terms of clarity, the proposed method achieves a much higher score of 90, compared to 65 for the NSCT method, indicating superior signal definition and quality. The noise level is reduced drastically, with the proposed method registering 15 dB, whereas NSCT only reaches 5 dB, showing better noise suppression. The frequency resolution also sees a major improvement, with the proposed method achieving 2 Hz compared to 10 Hz in the NSCT method, allowing for finer distinctions between frequency components. Energy scalograms, which represent energy distribution across time and frequency, also reflect a notable enhancement, with a score of 85 in the proposed method versus only 30 for NSCT, indicating better energy capture and representation. Additionally, the signal-to-noise ratio (SNR), a key measure of signal clarity, shows a dramatic increase from 1 dB with NSCT to 25 dB with the proposed method, highlighting the effectiveness of the new method in enhancing signal quality. Finally, when it comes to handling cyber anomalies, the proposed method scores 95, significantly higher than NSCT’s 60, suggesting that the proposed method is much more capable of detecting and mitigating cyber-related disruptions in signal processing. This table presents the performance metrics for Cyber Anomaly Detection, showing impressive results across various dimensions of signal processing. The Clarity score is high at 92, indicating that the method used produces clear and well-defined signals, which is essential for effective detection of cyber anomalies. The Noise Level is 20 dB, suggesting that despite the presence of noise, the detection method handles it well while maintaining clarity. The Frequency Resolution is very fine at 1 Hz, which means the method can precisely identify different frequency components, a critical aspect when distinguishing between normal signal patterns and cyber anomalies. The Energy Scalograms score of 90 reflects strong energy concentration across both time and frequency, indicating efficient tracking of signal characteristics over time. Additionally, the Signal-to-Noise Ratio (SNR) is excellent at 30 dB, which suggests that the method effectively enhances the signal quality in noisy environments. Lastly, the Handling Cyber Anomalies score of 95 demonstrates the method’s exceptional ability to detect and manage cyber anomalies, ensuring robust performance in maintaining signal integrity and security.

**Table 12:** Comparison of proposed method with other existing methods.

Metric	NSCT Method	Proposed Method (Wavelet-based Scalograms)	Cyber Anomaly Detection by proposed method
Clarity	Moderate clarity for standard disturbances.	High clarity, especially in high-frequency disturbances.	Clear identification of anomalies
Noise Handling	Poor handling of high-frequency noise.	Excellent noise suppression at multiple frequency levels.	Isolates cyber-induced noise clearly.
Frequency Resolution	Limited resolution, struggles with harmonics.	Higher frequency resolution for different levels.	Higher resolution for cyber anomalies.
Energy Scalograms (S)	Limited in distinguishing energy across bands.	Clear distribution of energy across time and frequency.	Enhanced energy detection of anomalies
Signal-to-Noise Ratio (SNR)	Lower SNR, less accurate for noisy signals.	Higher SNR, isolates disturbances with minimal noise.	Improved SNR for anomaly detection.
Handling Cyber Anomalies	Struggles to detect high-frequency anomalies.	Clear detection of cyber anomalies (e.g., FDI, harmonics).	Excellent for high-frequency anomalies

Table 12 compares the performance of two methods—Normal NSCT Method and Proposed Method in terms of signal processing metrics. The NSCT Method shows moderate clarity and limited frequency resolution, struggling with high-frequency noise and harmonics, and provides lower signal-to-noise ratio (SNR). In contrast, the Proposed Method excels in noise suppression and energy distribution, offering higher clarity, especially in high-frequency disturbances, and achieving a higher SNR. Cyber Anomaly Detection by proposed method further enhances these aspects by isolating and clearly identifying cyber-induced anomalies, delivering excellent clarity, frequency resolution, and energy scalogram performance, making it highly effective for detecting cyber anomalies like false data injection (FDI) and harmonic distortions.

Table 13 highlights the comparative performance of three methods: the NSCT Method, the Proposed Method (Wavelet-based Scalograms), and Cyber Anomaly Detection. The NSCT Method demonstrates moderate clarity and limited frequency resolution, struggling particularly with high-frequency noise and harmonics, resulting in a lower signal-to-noise ratio (SNR). The Proposed Method significantly improves upon this, offering excellent noise suppression, better energy distribution across frequencies, and higher clarity, especially for high-frequency disturbances, with an increased SNR. The Cyber Anomaly Detection method takes this a step further by effectively isolating and detecting cyber-induced anomalies, delivering superior clarity, frequency resolution, and enhanced energy scalograms, making it particularly adept at identifying cyber anomalies such as false data injection (FDI) and harmonic disturbances. Precision quantifies how many of the detected events are truly relevant, while recall measures the proportion of actual relevant events that were successfully detected. The F1-score, as the harmonic mean of precision and recall, is particularly important for evaluating performance in scenarios with class imbalance, which is common in power quality (PQ) disturbance datasets. Computational complexity provides insights into the practicality of implementing each method by considering factors such as training time, hardware requirements, and inference latency. By incorporating these additional metrics and methodological distinctions into the performance comparison, the evaluation becomes more comprehensive and meaningful. It not only highlights the robustness and accuracy of the proposed hybrid model but also provides a balanced perspective on the trade-offs involved, thereby ensuring a fair and multi-dimensional comparison with existing state-of-the-art approaches.

**Table 13:** Comparative evaluation of PQD detection Methods.

Method	Accuracy (%)	F1-Score (%)	Complexity	Key Highlights
Proposed (NSCT + SALSA + MCA)	**99.75**	**99.75**	Moderate (real-time, GPU-enabled)	Multi-resolution + sparse modelling + morphological filtering; highly noise-resilient
Ghaffarian & Mehregan (2020)^22^	98.50	98.00	Moderate	ML-based classification; limited robustness under noise
Saxena & Kumar (2019)^23^	97.90	97.65	Low	Wavelet-based; efficient but basic classifier
Chen & Xu (2021)^24^	97.20	96.90	Moderate	Harmonic analysis; less focused on classification
Yang & Tan (2022)^25^	98.80	98.40	High	Deep learning; strong results but needs large data
Wang & Zhang (2020)^26^	98.60	98.25	High	Wavelet + NN; accurate but moderate noise resilience
Tian & Song (2021)^27^	97.50	97.15	Moderate	Harmonic analysis in microgrids; waveform focus
Huang & Liu (2023)^28^	99.00	98.67	High	NN under noise; robust, but high computation demand
Xie & Zhang (2021)^29^	98.90	98.50	High	Hybrid method; effective but resource-intensive
Mitra & Gupta (2022)^30^	98.30	97.95	Moderate	Cyber-physical focus; PQ part of broader framework
Das & Basu (2018)^31^	97.80	97.40	Low	S-transform; simple, moderate noise sensitivity
Presekal et al. (2025)^32^	97.00	96.65	Moderate	PQ secondary to cyberattack study
Semertzis et al. (2024)^33^	97.40	97.05	Moderate	Focus on system stability; PQ part of analysis

**Table 14:** Comparative summary of Complexities.

Method	Time Complexity	Space Complexity
Proposed Method	$\:O\left(nlogn\right)$	$\:O\left(n\right)$
Standard NSCT	$\:O\left(nlogn\right)$	$\:O\left(n\right)$
Discrete Wavelet Transform	$\:O\left(n\right)$	$\:O\left(n\right)$
Short-Time Fourier Transform (STFT)	$\:O(n\cdot\:k)$	$\:O\left(n\right)$
Empirical Mode Decomposition (EMD)	$\:O\left(n2\right)$	$\:O\left(n\right)$

As per the Table 14 best method for efficiency is DWT stands out as the best method in terms of time complexity with $\:O\left(n\right)$ for both time and space, making it very efficient for smaller datasets. If considering methods that handle larger datasets, Proposed Method (Adaptive NSCT + SALSA + MCA) and Standard NSCT are favourable due to their logarithmic time complexity, especially when adaptive processing is beneficial for more complex data structures.

*F. Effect of Hardware Constraints (Sampling Rates and Sensor Inaccuracies)*:

In practical systems, measurement hardware (e.g., sensors and analog-to-digital converters) introduces constraints like limited sampling rates and sensor inaccuracies. These constraints affect both the time and frequency resolution of your analysis.

1. Sampling Rate Constraints:

The sampling rate fs​ determines the Nyquist frequency $\:{f}_{Nyquist}=\frac{{F}_{s}}{2}$, which limits the maximum frequency that can be captured without aliasing. For real-time systems, you need to ensure that the sampling rate is sufficiently high to capture the frequency content of the power quality disturbances of interest (such as harmonics, transients, and sags).nIf the sampling rate is too low, high-frequency disturbances may not be captured accurately, leading to aliasing. You can estimate the risk of aliasing using the Nyquist-Shannon Sampling Theorem (17):

20 $$\:{F}_{s}>2{f}_{max}$$

where $\:{f}_{max}$ is the highest frequency of interest in the disturbance. Ensuring that the sampling rate $\:{F}_{s}$meets this condition is vital for accurate signal capture.

2. Impact of Measurement Noise on the Method:

In real-world power systems, measurements often suffer from various types of noise (e.g., Gaussian noise, impulse noise, or quantization noise). When applying the Non-Subsampled Contourlet Transform (NSCT) or Sparse and Low-Rank Matrix Approximation (SALSA), it’s crucial to show how these noise types affect the analysis.

3. Noise Sensitivity Analysis:

To analyse the robustness of the method under noisy conditions, you should introduce a noise model into the input signal I(t), which can be represented as (21):

21 $$\:{I}_{\left\{\left\{noisy\right\}\right\}}\left(t\right)=\:I\left(t\right)+\:\varepsilon\left(t\right)\:$$

where: $\:{I}_{\left\{\left\{noisy\right\}\right\}}\left(t\right)$is the observed noisy signal. I(t) is the original, clean signal. ε(t) represents the noise component, which could be modelled as Gaussian or any other type of relevant noise for the application.

4.Signal-to-Noise Ratio (SNR):

To quantify the noise impact, define the Signal-to-Noise Ratio (SNR) of the noisy signal as:

22 $$\:SNR=\frac{\sum\:_{N=0}^{N-1}{\left({t}_{n}\right)}^{2}}{\sum\:_{N=0}^{N-1}{\left({\varepsilon}_{n}\right)}^{2}}$$

A higher SNR indicates less noise interference. By simulating noisy signals with different SNRs, you can analyse the sensitivity of the method to various noise levels.

*G.Decomposition Quality in the Presence of Noise*:

The NSCT decomposition and SALSA matrix approximation behave in the presence of noise. Energy of the noisy sub-bands $\:{D}_{k}$as:

23 $$\:{E}_{noisy}\left(S,\:\theta\:\right)=\sum\:_{n=0}^{N-1}{\left|{D}_{K}\left({t}_{n}\right)+\stackrel{-}{{\varepsilon}_{k}}\left({t}_{n}\right)\right|}^{2}$$

$\:{D}_{K}\left({t}_{n}\right)$) is the directional sub-band component at sample $\:{t}_{n}$. $\:\stackrel{-}{{\varepsilon}_{k}}\left({t}_{n}\right)$ represents the noise-induced components in the k-th sub-band.

This analysis helps quantify the degradation in decomposition quality and energy preservation when noise is introduced.

1. Sensor Inaccuracies:

Sensors in real power systems are subject to inaccuracies due to factors like calibration errors, drift, and quantization errors. These inaccuracies can be modelled as additive errors δ(t) in the measurement, which can affect both the magnitude and phase of the signal:

24 $$\:{I}_{measurement\left(t\right)}=\:I\left(t\right)+\:\delta\:\left(t\right)$$

where:$\:{I}_{measurement\left(t\right)}$is the signal as observed by the sensor, which includes measurement errors δ(t). The impact of sensor inaccuracies on the method can be evaluated by simulating the errors and observing the change in energy calculations and decomposition quality. For instance, you can compute the error in sub-band energy as (2.5):

Where:

25 $$\:{E}_{errors}\left(s,\theta\:\right)=\sum\:_{n=0}^{1}{\left\lceil{D}_{k}\left({t}_{n}\right)-{D}_{measument}\left({t}_{n}\right)\right\rceil}^{2}$$

$\:{D}_{measument}\left({t}_{n}\right)$ is the decomposition of the noisy or inaccurate signal.

## Conclusions

In conclusion, this research presents a comprehensive and innovative mathematical framework for enhancing feature extraction in power quality analysis through the integration of Non-Subsampled Contourlet Transform (NSCT), Split Augmented Lagrangian Shrinkage Algorithm (SALSA), and Morphological Component Analysis (MCA). The proposed approach significantly advances the detection and classification of power quality disturbances and cyber anomalies in modern electrical distribution systems, particularly within the context of smart grids and cyber-physical infrastructure. Despite its strengths, several limitations must be acknowledged. The complexity of the combined techniques demands considerable computational power and domain-specific expertise, potentially limiting widespread adoption, especially in resource-constrained environments. Furthermore, while the methodology is adaptable across a broad frequency range, its performance in highly dynamic or atypical grid conditions may require further parameter tuning, which could complicate deployment. The machine learning components of the framework rely heavily on diverse and representative datasets, which can be challenging to acquire, particularly in real-time monitoring scenarios. Additionally, as the grid continues to evolve, new forms of disturbances may emerge that are not fully addressed by the current model, highlighting the need for continuous refinement. To ensure practical applicability, real-time processing requirements must be considered. Efficient implementation of NSCT and SALSA in real-time environments necessitates optimizing computational loads by reducing the number of decomposition levels (s) and directional sub-bands (k), while parallelization and the use of hardware accelerators such as FPGAs or GPUs can enhance responsiveness. The robustness of the method should be validated through simulations incorporating different types of noise (e.g., Gaussian, impulse), reduced sampling rates, and sensor-related inaccuracies such as drift and quantization error. By analyzing standard power quality disturbances like harmonics, sags, and transients under these constraints, the framework’s performance can be evaluated in terms of signal energy preservation, fidelity of decomposition, and accuracy in detection. Overall, despite some implementation challenges, the proposed framework offers a scalable and intelligent solution for advancing fault detection and power quality analysis in complex, real-world power systems.

## Data Availability

Data Availability: The datasets used and/or analysed during the current study available from the corresponding author on reasonable request.
